# Enhancing Emotional Experience in AI-Enabled Robotics for Older Adults: A Kansei Engineering Approach

**DOI:** 10.1093/geroni/igaf122.2246

**Published:** 2025-12-31

**Authors:** Chorong Park, Rua Williams

**Affiliations:** Purdue University, West Lafayette, Indiana, United States; Purdue University, West Lafayette, Indiana, United States

## Abstract

As the global population ages, there is a growing need for technologies that not only assist older adults in their daily lives but also enhance their emotional well-being and social engagement. While assistive robots have been widely developed for functional support, their ability to foster meaningful human-robot interactions remains limited. This study explores how Kansei Engineering for Older Adults, a design methodology focused on emotional experience of older adults, can improve the way older adults engage with AI-enabled robotic companions. Through ethnographic research and interaction studies, we examine how four AI-powered robots, designed with Kansei Engineering principles—including facial expressions, voice modulation, and haptic feedback—affect social engagement and emotional connection in older adults. We measure engagement through interaction frequency, duration, and qualitative user feedback, providing insight into how emotional responsiveness in robots influences trust, companionship, and long-term acceptance. Preliminary findings suggest that older adults respond more positively to robots that express emotions naturally and adapt their interactions based on user feedback. Participants reported feeling more connected and engaged, highlighting the potential for emotionally intelligent robotics to reduce loneliness and improve quality of life. This study contributes to the growing field of human-centered AI and gerontechnology, offering a framework for designing emotionally aware robotic systems that foster social and psychological well-being in aging populations. As technology continues to evolve, integrating Kansei-driven AI interactions may shape the future of companion robotics, caregiving technology, and digital social engagement for older adults.

